# Supplementary material for: Ultrafast charge ordering by self-amplified exciton–phonon dynamics in TiSe2
Source: Nat Commun. 2020 Jan 2;11:43. doi: 10.1038/s41467-019-13672-7 (PMC6940384; doi:10.1038/s41467-019-13672-7)
Supplement: Supplementary file 1 — Supplementary Information [file 41467_2019_13672_MOESM1_ESM.pdf]

Supplementary Information for "Ultrafast charge ordering by self-amplified exciton-phonon dynamics in $TiSe_2$"

Chao Lian, Sheng-Jie Zhang, Shi-Qi Hu, Meng-Xue Guan, and Sheng Meng*
Beijing National Laboratory for Condensed Matter Physics and Institute of Physics, Chinese Academy of Sciences, Beijing, 100190, P. R. China
School of Physical Sciences, University of Chinese Academy of Sciences, Beijing, 100190, P. R. China and
Songshan Lake Materials Laboratory, Dongguan, Guangdong 523808, P. R. China
(Dated: November 13, 2019)

* smeng@iphy.ac.cn

SUPPLEMENTARY NOTE I: ALGORITHMS OF TDDFT IN PLANEWAVE BASIS

A. Planewave and Adiabatic Basis

Following our previous scenario in the Time dependent *ab initio* package (TDAP) [1–3], we implement the TDDFT algorithm in PW basis. The time-dependent Kohn-Sham (TDKS) equation at time t in PW basis $\{\mathbf{G}\}$ reads [4]:

$$i\hbar\frac{\partial\psi_{\gamma\mathbf{k}}(\mathbf{G},t)}{\partial t} = \mathcal{H}_{\mathbf{k}}(t)\psi_{\gamma\mathbf{k}}(\mathbf{G},t) \tag{1}$$

where $\psi_{\gamma\mathbf{k}}(\mathbf{G},t)$ is TDKS orbital, γ denotes the band index, $\mathbf{k}$ is the reciprocal momentum index. $\mathcal{H}_{\mathbf{k}}(t)$ is the Hamiltonian expanded with plane-wave basis, with matrix element

$$\begin{aligned}\mathcal{H}_{\mathbf{k}}(\mathbf{G},\mathbf{G}',t) =& T_{\mathbf{k}}(\mathbf{G},\mathbf{G}',t) + V(\mathbf{G},\mathbf{G}',t)\\ =& \frac{\hbar^2}{2m}|\mathbf{k}+\mathbf{G}+\mathbf{A}(t)|^2\delta_{\mathbf{G},\mathbf{G}'} + V(\mathbf{G},\mathbf{G}',t)\end{aligned} \tag{2}$$

where $T_{\mathbf{k}}(\mathbf{G},\mathbf{G}') = \frac{\hbar^2}{2m}|\mathbf{k}+\mathbf{G}+\mathbf{A}(t)|^2\delta_{\mathbf{G},\mathbf{G}'}$ is the kinetic term, $\mathbf{A}$ is the velocity gauge potential [5, 6]:

$$\mathbf{A}(t) = -\int_0^t \mathbf{E}(t')dt' \tag{3}$$

where $\mathbf{E}$ is electric field. $V(\mathbf{G},\mathbf{G}')$ is the potential term calculated within the corresponding module Quantum Espresso, including such as ion-electron potential, Hartree potential and exchange-correlation potential.

There are N_b independent TDKS equations for each $\mathbf{k}$ index, and thus $N_bN_{\mathbf{k}}$ equations in total, where N_b is the number of bands, typically, $N_{\mathbf{k}}$ is the number of $\mathbf{k}$ points. Typically, the orders of $N_{\mathbf{k}}$ and N_b are both $\sim 10^2$. The number of $\{\mathbf{G}\}$, N_G, is usually $\sim 10^4$. Thus, if the number of time steps is N_t, the complexity of solving Eq. (1) is $O(N_tN_bN_{\mathbf{k}}N_G^2)$.

We express the TDKS orbitals $\psi_{\gamma\mathbf{k}}(\mathbf{G},t)$ using adiabatic basis $\{\phi_{\gamma\mathbf{k}}(\mathbf{G},t_1)\}$

$$|\psi_{\gamma\mathbf{k}}(\mathbf{G},t)\rangle = \sum_i c_{i\gamma,\mathbf{k}}(t)\,|\phi_{i\mathbf{k}}(\mathbf{G},t_1)\rangle \tag{4}$$

where $c_{i\gamma,\mathbf{k}}(t)$ is the TD coefficients and the adiabatic basis $\{\phi_{i\mathbf{k}}\}(t_1)$ are solved by diagonalizing the Hamiltonian

$$\mathcal{H}_{\mathbf{k}}(\mathbf{G},t_1)\,|\phi_{i\mathbf{k}}(\mathbf{G},t_1)\rangle = \epsilon_{i\mathbf{k}}(t_1)\,|\phi_{i\mathbf{k}}(\mathbf{G},t_1)\rangle\,, \tag{5}$$

where the $\epsilon_{i\mathbf{k}}$ is the eigenvalue. The initial condition is chosen as

$$\begin{aligned}|\psi_{i\mathbf{k}}(\mathbf{G},t=0)\rangle :=& |\phi_{i\mathbf{k}}(\mathbf{G},t_1=0)\rangle\,,\\ c_{i\gamma,\mathbf{k}}(t=0) :=& \delta_{i\gamma},\end{aligned} \tag{6}$$

where $\delta_{i\gamma}$ is Kronecker delta. The TDKS equations are interpreted with the evolution of coefficient matrix

$$H_{\mathbf{k}}(t)C_{\mathbf{k}}(t) = i\hbar\frac{\partial}{\partial t}C_{\mathbf{k}}(t), \tag{7}$$

where

$$C_{\mathbf{k}}(t) = \begin{pmatrix} c_{11,\mathbf{k}}(t) & c_{12,\mathbf{k}}(t) & \cdots & c_{1N_b,\mathbf{k}}(t)\\ c_{21,\mathbf{k}}(t) & c_{12,\mathbf{k}}(t) & \cdots & c_{1N_b,\mathbf{k}}(t)\\ \vdots & \vdots & \cdots & \vdots\\ c_{N_b1,\mathbf{k}}(t) & c_{N_b2,\mathbf{k}}(t) & \cdots & c_{N_bN_b,\mathbf{k}}(t)\end{pmatrix}, \tag{8}$$

$$H_{\mathbf{k}}(t) = \begin{pmatrix} h_{11,\mathbf{k}}(t) & h_{12,\mathbf{k}}(t) & \cdots & h_{1N_b,\mathbf{k}}(t)\\ h_{21,\mathbf{k}}(t) & h_{12,\mathbf{k}}(t) & \cdots & h_{1N_b,\mathbf{k}}(t)\\ \vdots & \vdots & \cdots & \vdots\\ h_{N_b1,\mathbf{k}}(t) & h_{N_b2,\mathbf{k}}(t) & \cdots & h_{N_bN_b,\mathbf{k}}(t)\end{pmatrix}, \tag{9}$$

and $h_{ij,\mathbf{k}} = \langle\phi_{i\mathbf{k}}(t_1)|\mathcal{H}_{\mathbf{k}}(t)|\phi_{j\mathbf{k}}(t_1)\rangle$. To distinguish, we use $\mathcal{A}$ and A to represent the matrix A (e.g. Hamiltonian) in the PW basis and the adiabatic basis, respectively.

Note that, no approximation has yet been introduced in the derivation. The complexity of solving the Eq. 5 is the same as solving the Eq. 1, while it is less time consuming to solve Eq. 7 noticing that the dimension of $C_{\mathbf{k}}(t)$ and $H_{\mathbf{k}}(t)$ is $N_b \times N_b$ and $N_b \ll N_G$. The computation will be accelerated if the time of solving of Eq. 5 is minimized.

B. Efficient Evolution in Adiabatic Basis

An efficient scheme is raised by Wang *et. al.* [7]: Hamiltonian on adiabatic basis $h_{ij,\mathbf{k}}(t) = \langle \phi_{i\mathbf{k}}(t_1)|\mathcal{H}_\mathbf{k}(t)|\phi_{j\mathbf{k}}(t_1)\rangle$ changes approximately linearly within $[t_1, t_2]$ with $t_2 = t_1 + \Delta t$ fs and $\Delta t \sim 0.2$ fs [8]:

$$H_\mathbf{k}(t) = H_\mathbf{k}(t_1) + \frac{t - t_1}{t_2 - t_1}[H_\mathbf{k}(t_2) - H_\mathbf{k}(t_1)], \tag{10}$$

The matrix element $h_{ij,\mathbf{k}}$ is evaluated as

$$h_{ij,\mathbf{k}}(t_1) = \langle \phi_i(t_1)|\mathcal{H}_\mathbf{k}(t_1)|\phi_j(t_1)\rangle = \delta_{ij}\epsilon_{i,\mathbf{k}}(t_1), \tag{11}$$

and

$$\begin{aligned}
H_{ij,\mathbf{k}}(t_2) &= \langle \phi_{i,\mathbf{k}}(t_1)|\mathcal{H}_\mathbf{k}(t_2)|\phi_{j,\mathbf{k}}(t_1)\rangle \\
&= \langle \phi_{i,\mathbf{k}}(t_1)|\sum_l \phi_{l,\mathbf{k}}(t_2)\rangle \langle \phi_{l,\mathbf{k}}(t_2)|\mathcal{H}_\mathbf{k}(t_2)|\sum_m \phi_{m,\mathbf{k}}(t_2)\rangle \langle \phi_{m,\mathbf{k}}(t_2)|\phi_{j,\mathbf{k}}(t_1)\rangle \\
&= \sum_l \sum_m a_{il,\mathbf{k}}(t_1,t_2) a^*_{mj,\mathbf{k}}(t_1,t_2) \langle \phi_{l,\mathbf{k}}(t_2)|\mathcal{H}_\mathbf{k}(t_2)|\phi_{m,\mathbf{k}}(t_2)\rangle \\
&= \sum_l \sum_m a_{il,\mathbf{k}}(t_1,t_2)\delta_{lm}\epsilon_{l\mathbf{k}}(t_2) a_{il,\mathbf{k}}(t_1,t_2) a^*_{mj,\mathbf{k}}(t_1,t_2) \\
&= \sum_l a_{il,\mathbf{k}}(t_1,t_2) a^*_{lj,\mathbf{k}}(t_1,t_2)\epsilon_{l\mathbf{k}}(t_2),
\end{aligned} \tag{12}$$

where

$$a_{il,\mathbf{k}}(t_1,t_2) = \langle \phi_{i,\mathbf{k}}(t_1)|\phi_{l,\mathbf{k}}(t_2)\rangle \tag{13}$$

$|\phi_{l,\mathbf{k}}(t_2)\rangle$ and $\epsilon_{l\mathbf{k}}(t_2)$ are the adiabatic basis and the eigenvalue at time t_2, which are solved in a self-consistent process, see detail at Sec. C .

Propagator operator $U_\mathbf{k}(t_2, t_1)$ is calculated with the knowledge of $H_\mathbf{k}(t)$ in the Crank-Nicholson scheme

$$U_\mathbf{k}(t_2,t_1) = \prod_{s=0}^{N_t} \frac{\exp[-i\hbar H(t_1 + sdt)dt/2]}{\exp[i\hbar H(t_1 + sdt)dt/2]} = \prod_{s=0}^{N_t} \frac{1 - i\hbar H_\mathbf{k}(t_1 + sdt)dt/2}{1 + i\hbar H_\mathbf{k}(t_1 + sdt)dt/2}, \tag{14}$$

where $dt = \Delta t/N_t$ is the integration time step. Since $dtH \ll 1$ is needed to satisfy the condition of $\exp(-i\hbar dtH) = 1 - i\hbar H dt$, we choose $dt \sim 0.1$ attosecond, considering H is about 10^2 eV. The coefficients $c_{i\gamma,\mathbf{k}}$ are thus propagated as

$$C_\mathbf{k}(t_2) = U_\mathbf{k}(t_2,t_1)C_\mathbf{k}(t_1). \tag{15}$$

Thus, we finish the evolution of TDKS orbital from t_1 to t_2 as

$$\begin{aligned}
|\psi_{\gamma\mathbf{k}}(t_2)\rangle &= \sum_i c_{i\gamma,\mathbf{k}}(t_2)\,|\phi_{i,\mathbf{k}}(t_1)\rangle \\
&= \sum_i c_{i\gamma,\mathbf{k}}(t_2) \sum_l |\phi_{l,\mathbf{k}}(t_2)\rangle \langle \phi_{l,\mathbf{k}}(t_2)|\phi_{i,\mathbf{k}}(t_1)\rangle \\
&= \sum_l \sum_i a^*_{il,\mathbf{k}}(t_1,t_2) c_{i\gamma,\mathbf{k}}(t_2)\,|\phi_{l,\mathbf{k}}(t_2)\rangle \\
&= \sum_l c'_{l\gamma,\mathbf{k}}(t_2)\,|\phi_{l,\mathbf{k}}(t_2)\rangle
\end{aligned} \tag{16}$$

where

$$c'_{l\gamma,\mathbf{k}}(t_2) = \sum_i a^*_{il,\mathbf{k}}(t_1,t_2) c_{i\gamma,\mathbf{k}}(t_2). \tag{17}$$

Charge density $\rho(t_2)$ can be calculated with $c'_{l\gamma,\mathbf{k}}(t_2)$ and $|\phi_{l,\mathbf{k}}(t_2)\rangle$ as

$$\begin{aligned}\rho(\mathbf{G},t_2) &= \sum_{\mathbf{k}}\sum_{\gamma}|\psi_{\gamma,\mathbf{k}}(\mathbf{G},t_2)|^2 \\ &= \sum_{\mathbf{k}}\sum_{\gamma}\sum_{i}|c'_{i\gamma,\mathbf{k}}(t_2)|^2\,|\phi_{i,\mathbf{k}}(\mathbf{G},t_2)|^2 \\ &= \sum_{\mathbf{k}}\sum_{i}\left[\sum_{\gamma}|c'_{i\gamma,\mathbf{k}}(t_2)|^2\right]|\phi_{i\mathbf{k}}(\mathbf{G},t_2)|^2 \\ &= \sum_{\mathbf{k}}\sum_{i}q_{i\mathbf{k}}(t_2)\,|\phi_{i\mathbf{k}}(\mathbf{G})|^2\,,\end{aligned} \tag{18}$$

where

$$q_{i\mathbf{k}}(t_2) = \sum_{\gamma}|c'_{i\gamma,\mathbf{k}}(t_2)|^2 \tag{19}$$

is the population of the adiabatic states.

C. Computational Flowchart

We organized the equations in a flowchart, as shown in Supplementary Fig. 1. Step (A) (B) (C) (H) (I) (K) are computed with the Quantum Espresso modules. As mentioned above, the algorithm requires foreknowledge of $\rho(\mathbf{G},t_2)$ in the calculation of Hamiltonian $H_{\mathbf{k}}(t)$. It is satisfied with a self-consistent process:

1. An initial guess of $\rho(\mathbf{G},t_2)$ is built using extrapolation from previous step.
2. Hamiltonian $H_{\mathbf{k}}(t)$ and propagator $U_{\mathbf{k}}(t_2,t_1)$ are built from $\rho(\mathbf{G},t_2)$ using Eq. 10 and Eq. 14, respectively.
3. An new $\rho(\mathbf{G},t_2)$ is calculated with new population propagated from Eq. 15.
4. The first three steps are repeated until the new and old $\rho(\mathbf{G},t_2)$ are the same.

D. Forces and Dynamics

Once the self-consistency in charge density evolution is satisfied, post-processing including the calculation of total energy, Hellmann-Feynman forces, and the ionic trajectory are invoked. For instance, the forces acting on the ions can be calculated through

$$\mathbf{F}_{\mathbf{R}_I} = \sum_{i\mathbf{k}}\langle\psi_{i\mathbf{k}}|\nabla_{\mathbf{R}_I}\mathcal{H}|\psi_{i\mathbf{k}}\rangle\,, \tag{20}$$

where $\mathbf{R}_I$ and $\mathbf{F}_{\mathbf{R}_I}$ are the position and force of Ith ion.

With $\mathbf{R}_I$ and $\mathbf{F}_{\mathbf{R}_I}$, we utilize the Ehrenfest theorem for evolving ions according to the equation of motion

$$M_I\frac{d^2\mathbf{R}_I}{dt^2} = \mathbf{F}_{\mathbf{R}_I}, \tag{21}$$

where M_I is the mass of Ith ion. The velocity $v_I(t) = d\mathbf{R}_I/dt$ and the temperature $T(t) = \sum_I^{N_I} M_I v_I^2(t)/2N_I$ are also calculated, where N_I is the total number of ions.

Besides the conventional NVE ensemble, additional thermostats, such as Nosé-Hoover [9, 10] and Berendsen [11] is considered to simulate different environmental conditions. In the damped MD simulations, we utilize a simple velocity-rescaling thermostat. The ionic velocities are rescaled at each time step as

$$\mathbf{v}'_I(t) = \mathbf{v}_I(t)\sqrt{T'(t)/T(t)}, \tag{22}$$

where $\mathbf{v}'_I(t)$ and $T'(t) = T(t) - \Delta T$ are the rescaled velocity and temperature, respectively. The decreasing rate $\Delta T = 0.01$ eV atom^{-1} ps^{-1} is used in the simulations.

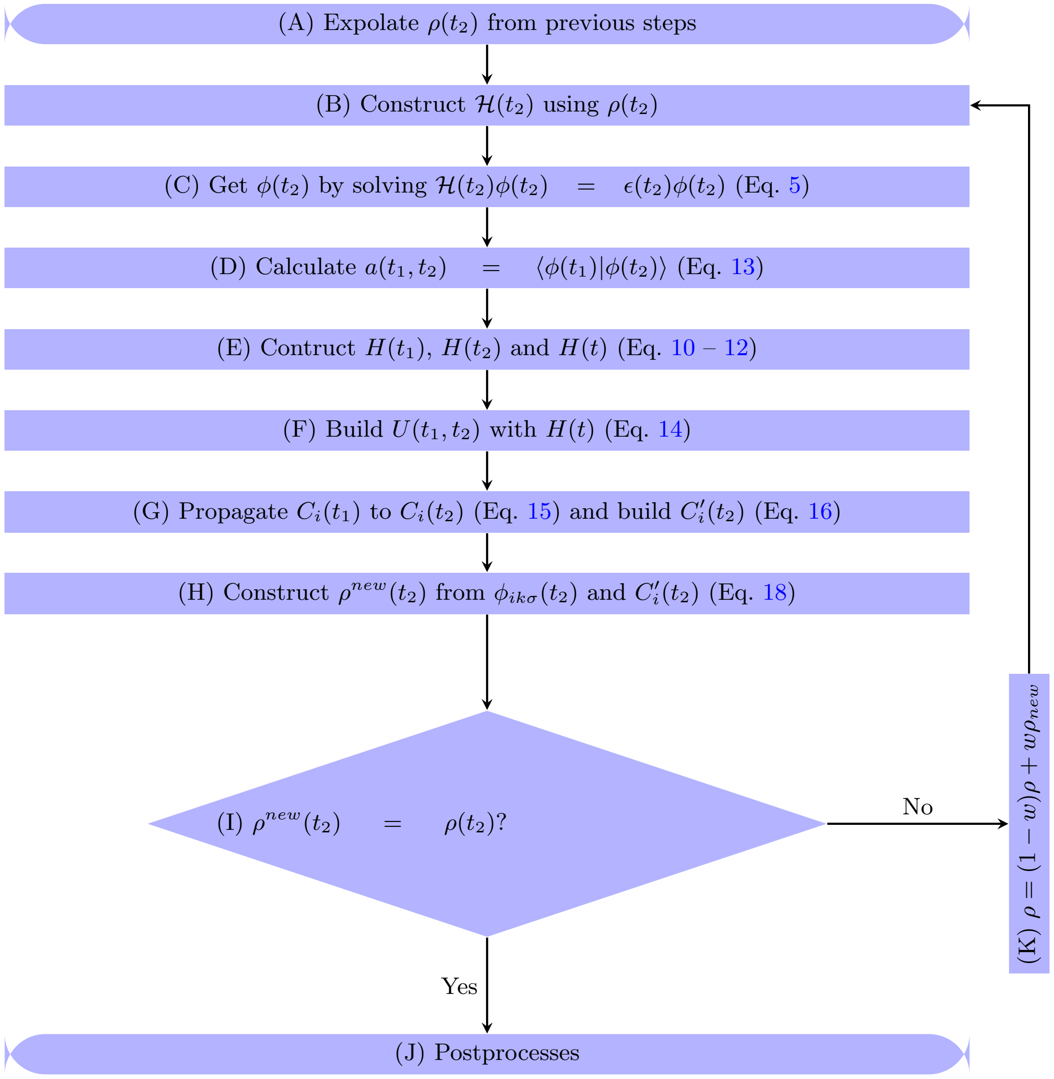

Supplementary Fig. 1. Flowchart of TDDFT algorithm.

Combining the TDKS equation and the Ehrenfest theorem, the many-body electron-electron interaction and the ionic movement under the excited-state TDKS wavefunction evolution are described in an *ab initio* way. We expect that the electron-electron interactions at the adiabatic XC level and electron-phonon scatterings within the mean-field average trajectory are present in these simulations. The excess electronic energy could dissipate into available phonon modes via electron-phonon coupling or to low-energy electrons via electron-electron scattering, resulting in carrier thermalization and cooling effect.

E. Projector Augmented Wave Method

To expand the TDKS orbital, the adiabatic basis $|\phi_{i\mathbf{k}}(\mathbf{G}, t_1)\rangle$ should be orthonormal. Eigenstates in all electron method and norm-conserving pseudopotential satisfy the orthonormal requirement naturally. However, using projector augmented-waves method (PAW) [12] bring additional core functions, which lead to nonorthogonal eigenstates. The formalism of DFT quantities such as total energy and forces using PAW method are described in Blöchl's original paper [12]. Here, we only list the related changes in TDDFT evolution.

Using PAW method, all the coefficients $C'_{\mathbf{k}}(t)$ are related with the pseudo-eigenstates $|\tilde{\phi}_{i\mathbf{k}}(\mathbf{G}, t_1)\rangle$

$$|\phi_{i\mathbf{k}}(\mathbf{G}, t_1)\rangle = \hat{T}\,|\tilde{\phi}_{i\mathbf{k}}(\mathbf{G}, t_1)\rangle \tag{23}$$

where $\hat{T}$ is the transform operator. The only change caused by PAW method is Eq (13). Using PAW, eq (13) becomes

$$\begin{aligned} a_{il,\mathbf{k}}(t_1,t_2) &= \langle\tilde{\phi}_{i\mathbf{k}}(t_1)|\hat{T}^\dagger\hat{T}|\tilde{\phi}_{l\mathbf{k}}(t_2)\rangle \\ &= \langle\tilde{\phi}_{i\mathbf{k}}(t_1)|\hat{S}|\tilde{\phi}_{l\mathbf{k}}(t_2)\rangle \end{aligned} \tag{24}$$

where

$$\hat{S} = \begin{pmatrix} s_{11,\mathbf{k}}(t) & s_{12,\mathbf{k}}(t) & \cdots & s_{1N_b,\mathbf{k}}(t) \\ s_{21,\mathbf{k}}(t) & s_{12,\mathbf{k}}(t) & \cdots & s_{1N_b,\mathbf{k}}(t) \\ \vdots & \vdots & \cdots & \vdots \\ s_{N_b1,\mathbf{k}}(t) & s_{N_b2,\mathbf{k}}(t) & \cdots & s_{N_bN_b,\mathbf{k}}(t) \end{pmatrix}, \tag{25}$$

$s_{ij,\mathbf{k}} = \langle\beta_{i\mathbf{k}}(\mathbf{G})|\phi_{j\mathbf{k}}(\mathbf{G})\rangle$, and $\beta_{i\mathbf{k}}(\mathbf{G})$ is the Kleinman-Bylander projectors [13].

F. Time Dependent Band Unfolding

The CDW phase of $TiSe_2$ is a 2×2 cell of the normal phase. The energy bands are folded from 1×1 Brillouin zone (BZ) to the 2×2 BZ. In contrast, ARPES measurements still span over the 1×1 BZ. To bridge the gap between DFT bands and measured ARPES spectra, the band unfolding technique is used to calculate the effective band structure (EBS) of the supercell (SC). Expanding the adiabatic basis $|\phi_{i,\mathbf{k}}(t)\rangle$ of 2×2 SC in the adiabatic basis $|\Phi_{I,\mathbf{K}}(t)\rangle$ of primitive 1×1 cell (PC), we get

$$|\phi_{i,\mathbf{k}}(\mathbf{G},t)\rangle = \sum_{I,\mathbf{K}} a(I,\mathbf{K};i,\mathbf{k};t)\,|\Phi_{I,\mathbf{K}}\mathbf{G},t)\rangle\,, \tag{26}$$

where $\mathbf{K} = \mathbf{k} + \mathbf{B}$ and $\mathbf{B}$ is the reciprocal basis vector of SC. The spectral function is the EBS along the $\mathbf{K}$ path in PCBZ [14, 15]:

$$A(\mathbf{K},E,t) = \sum_i P(\mathbf{K};\mathbf{k},i;t)\delta(E-\epsilon_{i,\mathbf{k}}(t)), \tag{27}$$

where E is the energy and

$$P(\mathbf{K};\mathbf{k},i) = \sum_I a^*(\mathbf{K},I;\mathbf{k},i;t)a(\mathbf{K},I;\mathbf{k},i;t) = \sum_{\mathbf{G}} |\phi_{i,\mathbf{k}}(\mathbf{G}+\mathbf{K}-\mathbf{k},t)|^2\,, \tag{28}$$

We can introduce an extra weight function $w(i,\mathbf{k})$

$$A(\mathbf{K},E,t) = \sum_i P(\mathbf{K};\mathbf{k},i;t)w_{i,\mathbf{k}}(t)\delta(E-\epsilon_{i,\mathbf{k}}(t)). \tag{29}$$

The choice of $w_{i,\mathbf{k}}$ is arbitrary [16]. Here, we use the population of the adiabatic states as the

$$w_{i,\mathbf{k}}(t) = q_{i,\mathbf{k}}(t) \tag{30}$$

to reproduce the intensity in ARPES spectra.

SUPPLEMENTARY NOTE II: FLUENCE DEPENDENCE OF PHOTOCARRIER DENSITY

We investigate the photo-carrier density as a function of time and laser fluence $n(t,I)$. Here, the carrier density is calculated as $n(t) = \frac{1}{2}\sum_{i,\mathbf{k}}|q_{i\mathbf{k}}(t) - q_{i\mathbf{k}}(t=0)|$. We note that $n(t)$ accurately describes the number of excited carriers after the laser field ends $A(t) = 0$. Otherwise, a gauge independent projection on $\psi_{k'}(t)$ with $k' = k - A(t)/c$ instead of $k' = k$ can be used [18–26]. These two projections are identical when the laser field ends $A(t) = 0$.

As shown in Supplementary Fig. 2, the $n(t = 20\text{ fs}, I)$ are approximately proportional to the laser fluences. After comparing with the experimental measurements [17], we find the $n(I)$ relations are consistent at both the low fluence area $I < 0.04$ mJ cm^{-2} and the high fluence area $I > 0.22$ mJ cm^{-2}, while a clear derivation from linearity is observed in experiments. Exciton correlation drives the superlinear feature but is underestimated in DFT/TDDFT calculations

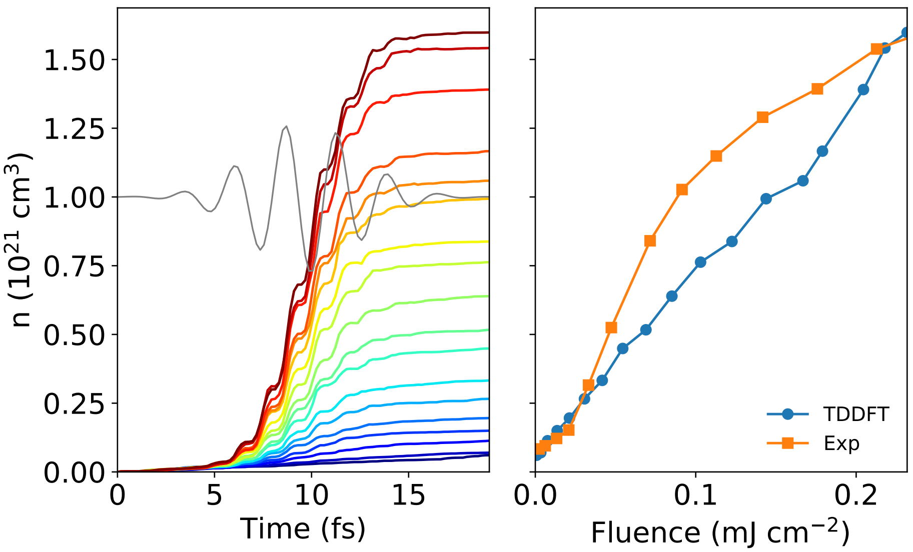

Supplementary Fig. 2. Photo-induced carrier density as a function of time (left panel) and a function of laser fluence (right panel). The grey line denotes the time-dependence of the laser pulse. The experimental data are reproduced from Ref. [17].

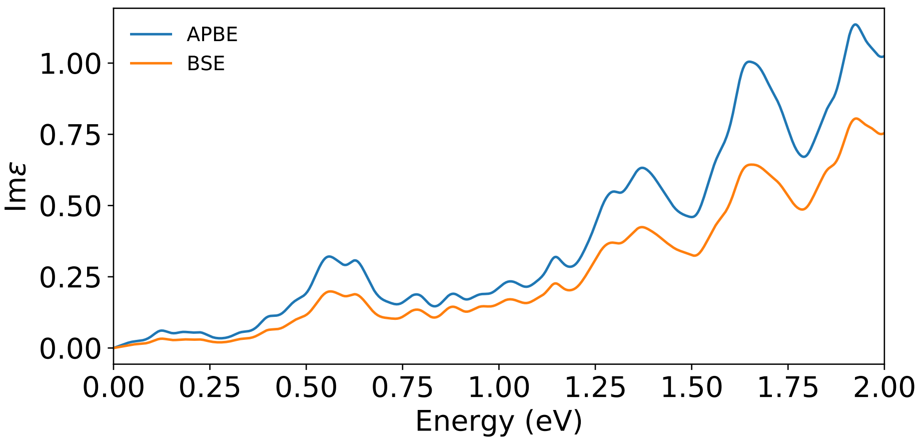

Supplementary Fig. 3. The imaginary part of the dielectric function of $TiSe_2$ at the momentum $\mathbf{q} = \mathbf{w}$ calculated from linear-response TDDFT with adiabatic PBE (APBE) and BSE kernels.

(Supplementary Fig. 3). Nevertheless, since the difference is minimized when $I > 0.22$ mJ cm^{-2}, we focus on the experimental phenomena in high laser fluence region, to reproduce the similar excitation states for direct comparisons.

Consistent with experimental observations [27], we note that the PLD dynamics are not sensitive to the photon energies. This is because that the band gap of CDW 1T-$TiSe_2$ (0.18 eV) is smaller than the photon energy in most commonly used laser sources (∼1 eV). Besides, the sub-picosecond laser pulses utilized in the experiments and our simulations would bring up significant broadening in photon energy as well as multi-photon absorption processes.

[1] Meng, S. & Kaxiras, E. Real-time, local basis-set implementation of time-dependent density functional theory for excited state dynamics simulations. *J. Chem. Phys.* **129**, 054110 (2008).
[2] Lian, C., Hu, S.-Q., Guan, M.-X. & Meng, S. Momentum-resolved TDDFT algorithm in atomic basis for real time tracking of electronic excitation. *J. Chem. Phys.* **149**, 154104 (2018).
[3] Lian, C., Guan, M., Hu, S., Zhang, J. & Meng, S. Photoexcitation in Solids: First-Principles Quantum Simulations by

Real-Time TDDFT. *Advanced Theory and Simulations* **1**, 1800055 (2018). URL https://onlinelibrary.wiley.com/doi/full/10.1002/adts.201800055.
[4] Runge, E. & Gross, E. K. U. Density-Functional Theory for Time-Dependent Systems. *Phys. Rev. Lett.* **52**, 997 (1984).
[5] Bertsch, G. F., Iwata, J.-I., Rubio, A. & Yabana, K. Real-space, real-time method for the dielectric function. *Phys. Rev. B* **62**, 7998 (2000).
[6] Yabana, K., Nakatsukasa, T., Iwata, J.-I. & Bertsch, G. F. Real-time, real-space implementation of the linear response time-dependent density-functional theory. *Phys. Status Solidi B* **243**, 1121 (2006).
[7] Wang, Z., Li, S.-S. & Wang, L.-W. Efficient Real-Time Time-Dependent Density Functional Theory Method and its Application to a Collision of an Ion with a 2D Material. *Phys. Rev. Lett.* **114**, 063004 (2015).
[8] Ren, J., Vukmirović, N. & Wang, L.-W. Nonadiabatic molecular dynamics simulation for carrier transport in a pentathiophene butyric acid monolayer. *Phys. Rev. B* **87**, 205117 (2013).
[9] Nosé, S. A unified formulation of the constant temperature molecular dynamics methods. *J. Chem. Phys.* **81**, 511–519 (1984).
[10] Hoover, W. G. Canonical dynamics: Equilibrium phase-space distributions. *Phys. Rev. A* **31**, 1695–1697 (1985).
[11] Berendsen, H. J. C., Postma, J. P. M., van Gunsteren, W. F., DiNola, A. & Haak, J. R. Molecular dynamics with coupling to an external bath. *J. Chem. Phys.* **81**, 3684–3690 (1984).
[12] Blchl, P. E. Projector augmented-wave method. *Phys. Rev. B* **50**, 17953 (1994).
[13] Giannozzi, P. *et al.* QUANTUM ESPRESSO: a modular and open-source software project for quantum simulations of materials. *J. Phys. Condens. Matter* **21**, 395502 (2009).
[14] Popescu, V. & Zunger, A. Extracting E versus k effective band structure from supercell calculations on alloys and impurities. *Phys. Rev. B* **85**, 085201 (2012).
[15] Medeiros, P. V. C., Stafström, S. & Björk, J. Effects of extrinsic and intrinsic perturbations on the electronic structure of graphene: Retaining an effective primitive cell band structure by band unfolding. *Phys. Rev. B* **89**, 041407 (2014).
[16] Lian, C. & Meng, S. Dirac cone pairs in silicene induced by interface Si-Ag hybridization: A first-principles effective band study. *Phys. Rev. B* **95**, 245409 (2017).
[17] Porer, M. *et al.* Non-thermal separation of electronic and structural orders in a persisting charge density wave. *Nat. Mater.* **13**, 857 (2014).
[18] Otobe, T. *et al.* First-principles electron dynamics simulation for optical breakdown of dielectrics under an intense laser field. *Phys. Rev. B* **77**, 165104 (2008).
[19] Otobe, T., Yabana, K. & Iwata, J.-I. First-principles calculation of the electron dynamics in crystalline SiO2. *J. Phys. Condens. Matter* **21**, 064224 (2009).
[20] Otobe, T., Shinohara, Y., Sato, S. A. & Yabana, K. Femtosecond time-resolved dynamical Franz-Keldysh effect. *Phys. Rev. B* **93**, 045124 (2016).
[21] Yabana, K., Sugiyama, T., Shinohara, Y., Otobe, T. & Bertsch, G. F. Time-dependent density functional theory for strong electromagnetic fields in crystalline solids. *Phys. Rev. B* **85**, 045134 (2012).
[22] Shinohara, Y. *et al.* First-principles description for coherent phonon generation in diamond. *J. Phys. Condens. Matter* **22**, 384212 (2010).
[23] Shinohara, Y. *et al.* Coherent phonon generation in time-dependent density functional theory. *Phys. Rev. B* **82**, 155110 (2010).
[24] Shinohara, Y. *et al.* Nonadiabatic generation of coherent phonons. *J. Chem. Phys.* **137**, 22A527 (2012).
[25] Sato, S. A. *et al.* Time-dependent density functional theory of high-intensity short-pulse laser irradiation on insulators. *Phys. Rev. B* **92**, 205413 (2015).
[26] Sato, S. A., Taniguchi, Y., Shinohara, Y. & Yabana, K. Nonlinear electronic excitations in crystalline solids using meta-generalized gradient approximation and hybrid functional in time-dependent density functional theory. *J. Chem. Phys.* **143**, 224116 (2015).
[27] Mhr-Vorobeva, E. *et al.* Nonthermal Melting of a Charge Density Wave in $TiSe_2$. *Phys. Rev. Lett.* **107**, 036403 (2011).
